# Validation of a five-level triage system in pediatric trauma and the effectiveness of triage nurse modification: A multi-center cohort analysis

**DOI:** 10.3389/fmed.2022.947501

**Published:** 2022-11-01

**Authors:** Tien-Tien Liu, Chi-Tung Cheng, Chih-Po Hsu, Chung-Hsien Chaou, Chip-Jin Ng, Mei-Jy Jeng, Yu-Che Chang

**Affiliations:** ^1^Department of Nursing, Chang Gung Memorial Hospital, Taoyuan, Taiwan; ^2^Institute of Emergency and Critical Care Medicine, National Yang-Ming Chiao-Tung University, Taipei, Taiwan; ^3^Division of Trauma and Emergency Surgery, Department of General Surgery, Chang Gung Memorial Hospital, Taoyuan, Taiwan; ^4^College of Medicine, Chang Gung University, Taoyuan, Taiwan; ^5^Chang Gung Medical Education Research Centre (CG-MERC), Taoyuan, Taiwan; ^6^Department of Emergency Medicine, Chang Gung Memorial Hospital, Taoyuan, Taiwan; ^7^National Working Group of Taiwan Triage and Acuity Scale (TTAS), Taipei, Taiwan; ^8^Department of Pediatrics, Taipei Veterans General Hospital, Taipei, Taiwan

**Keywords:** nurses, Taiwan, triage, Taiwan triage acuity scale, pediatrics, emergency department, trauma

## Abstract

**Introduction:**

Triage is one of the most important tasks for nurses in a modern emergency department (ED) and it plays a critical role in pediatric trauma. An appropriate triage system can improve patient outcomes and decrease resource wasting. However, triage systems for pediatric trauma have not been validated worldwide. To ensure clinical reliability, nurses are allowed to override the acuity level at the end of the routine triage process. This study aimed to validate the Taiwan Triage and Acuity Scale (TTAS) for pediatric trauma and evaluate the effectiveness of triage nurse modification.

**Methods:**

This was a multicenter retrospective cohort study analyzing triage data of all pediatric trauma patients who visited six EDs across Taiwan from 2015 to 2019. Each patient was triaged by a well-trained nurse and assigned an acuity level. Triage nurses can modify their acuity based on their professional judgment. The primary outcome was the predictive performance of TTAS for pediatric trauma, including hospitalization, ED length of stay, emergency surgery, and costs. The secondary outcome was the accuracy of nurse modification and the contributing factors. Multivariate regression was used for data analysis. The Akaike information criterion and C-statistics were utilized to measure the prediction performance of TTAS.

**Results:**

In total, 45,364 pediatric patients were included in this study. Overall mortality, hospitalization, and emergency surgery rates were 0.17, 5.4, and 0.76%, respectively. In almost all cases (97.48%), the triage nurses agreed upon the original scale. All major outcomes showed a significant positive correlation with the upgrade of acuity levels in TTAS in pediatric trauma patients. After nurse modification, the Akaike information criterion decreased and C-statistics increased, indicating better prediction performance. The factors contributing to this modification were being under 6 years of age, heart rate, respiratory rate, and primary location of injuries.

**Conclusion:**

The TTAS is a reliable triage tool for pediatric trauma patients. Modification by well-experienced triage nurses can enhance its prediction performance. Younger age, heart rate, respiratory rate, and primary location of injuries contributed to modifications of the triage nurse. Further external validation is required to determine its role in pediatric trauma worldwide.

## Introduction

According to the Global Burden of Disease 2017 study, an estimated 1,900 children and adolescents die every day because of injuries (1). Traumatic injury is the leading cause of mortality and disability, which is considered as a significant public health issue among children worldwide (2). Trauma care is highly time-sensitive, and the early identification of potentially fatal injuries and conditions is crucial for survival (3, 4). Therefore, triage is a critical component of trauma care, which is defined as the process of rapidly and accurately prioritizing patients and assigning them to appropriate levels of urgency for initiating treatment and investigations (5). The accuracy of triage systems ultimately determines the quality of care the patient needs and receives (6). Previous studies that focused on the effectiveness of triage scales in emergency departments (EDs) are mainly conducted among non-trauma pediatric patients (7, 8) or in the pre-hospital settings (9). Currently, no triage systems have been explicitly validated for pediatric trauma patients presenting to EDs.

Performing triage and assessment for children in an emergency medicine context seems more challenging than it is for adults. Several studies have shown that special considerations are required in the triage process because of developmental, epidemiological, and physiological variations at different ages (10–13). First, a principal challenge is the judgment or modification of the parameters obtained in the first contact with the triage nurse as abnormal vital signs based on the wide reference range of normal heart rate (HR) and respiratory rate (RR) in different age populations. Designation of higher acuity levels solely based on abnormal vital signs might lead to inappropriate triage to a higher acuity, namely, over-triage, which may delay care delivery to other potentially more urgent patients (14). Chang et al. observed that vital signs, particularly HRs, were the primary reason for over-triage in young children (15, 16). Secondly, one of the challenges in pediatric triage is that the anatomical and physiological differences between adults and children imply that children are not just adults on a smaller scale (9, 17). Children have less fat and more connective tissue covering a flexible skeleton, protecting packed abdominal and thoracic structures (18). As a result, their bodies will have different responses to different injury mechanisms than adults. For instance, children's rib cages are more flexible than those of adults. Rib fractures are commonly seen in adults with minor chest trauma; however, rib fractures indicate severe chest trauma in children. Other challenges in pediatric triage are communication difficulties and the strong confounding effects of emotional responses to unfamiliar environments, especially for children under 2 years of age (19, 20). When recording their medical history, young children also have difficulty expressing their feelings or complaints accurately and often start crying and become uncooperative or irritable due to pain or fright. Thus, vital signs, injury patterns, and injury mechanisms have been considered essential modifiers in combination with pediatric patients' major clinical presentations in the current pediatric triage system. As mentioned above, it is a complex and challenging task for triage nurses to develop or conduct a triage system with acceptable accuracy for all ages in the ED. Training triage nurses to accurately perform assessment skills in pediatric trauma patients at triage, and are effectively assisted by a validated pediatric triage system, can determine the quality of acute care. A previous study showed that the subjective modification of the initial triage level by experienced personnel could lead to a more accurate prediction of disposition (14).

Apart from the vitals of pediatric trauma patients, to our knowledge, no study has explicitly explored the effectiveness of the acuity level modified by triage nurses and investigated the factors that contributed to the modification. This study aimed to examine the effectiveness of Taiwan's five-level triage system, the Taiwan Triage and Acuity Scale (TTAS), in acute pediatric trauma patients and compare the predictive performance of triage systems before and after modification by triage nurses. Additionally, our study can add new understanding and evidence for pediatric trauma patient triage amendments.

## Methods

### Study design and setting

This multicenter retrospective cohort study was conducted to analyze the data of pediatric (age <18 years) trauma patients who visited the six EDs in the largest healthcare system in Taiwan from January 2015 to December 2019. We included two large urban academic medical centers with an average annual ED census of approximately 15,000 and 10,000 pediatric trauma patients, two regional hospitals with an average of approximately 6,800 and 5,930 annual pediatric trauma ED visits, and two district hospitals with an average of 5,300 and 1,400 pediatric trauma patients in the ED per year. All medical records of the patients were reviewed from the Chang Gung Research Database, which is the most extensive multi-institutional electronic medical record collection in Taiwan, and contains all necessary records from every visiting patient, including details of their triage information, vital signs, treatments, clinical outcomes, and daily medical records of doctor and nursing staff (21). All data are de-identified and encrypted to protect the participants' privacy.

Each pediatric patient was triaged by a triage nurse using the computerized version of the TTAS, which was developed in strict compliance with the TTAS standard and provided nurses with the support of quick access to the standardized complaints list and allocation of an appropriate triage level. Well-trained triage nurses are allowed to override the acuity level for each patient at the end of the routine triage process of TTAS if the computer-generated triage score does not correspond to the nurses' clinical impression. However, the reason for this override must be recorded. Physicians see pediatric patients based on the final triage levels assigned after the modification; however, the initial levels are also available to physicians in triage notes. All triage nurses with more than 3 years of experience in the ED had undergone standardized training on the TTAS for at least 3 months and were certified by triage nurse educators. This study was approved by the Institutional Review Board of Chung Gang Memorial Hospital (IRB number 2004110008). Owing to the retrospective design and use of anonymous data, informed consent was not considered necessary for pediatric patients.

### Selection of participants

**Figure 1:**
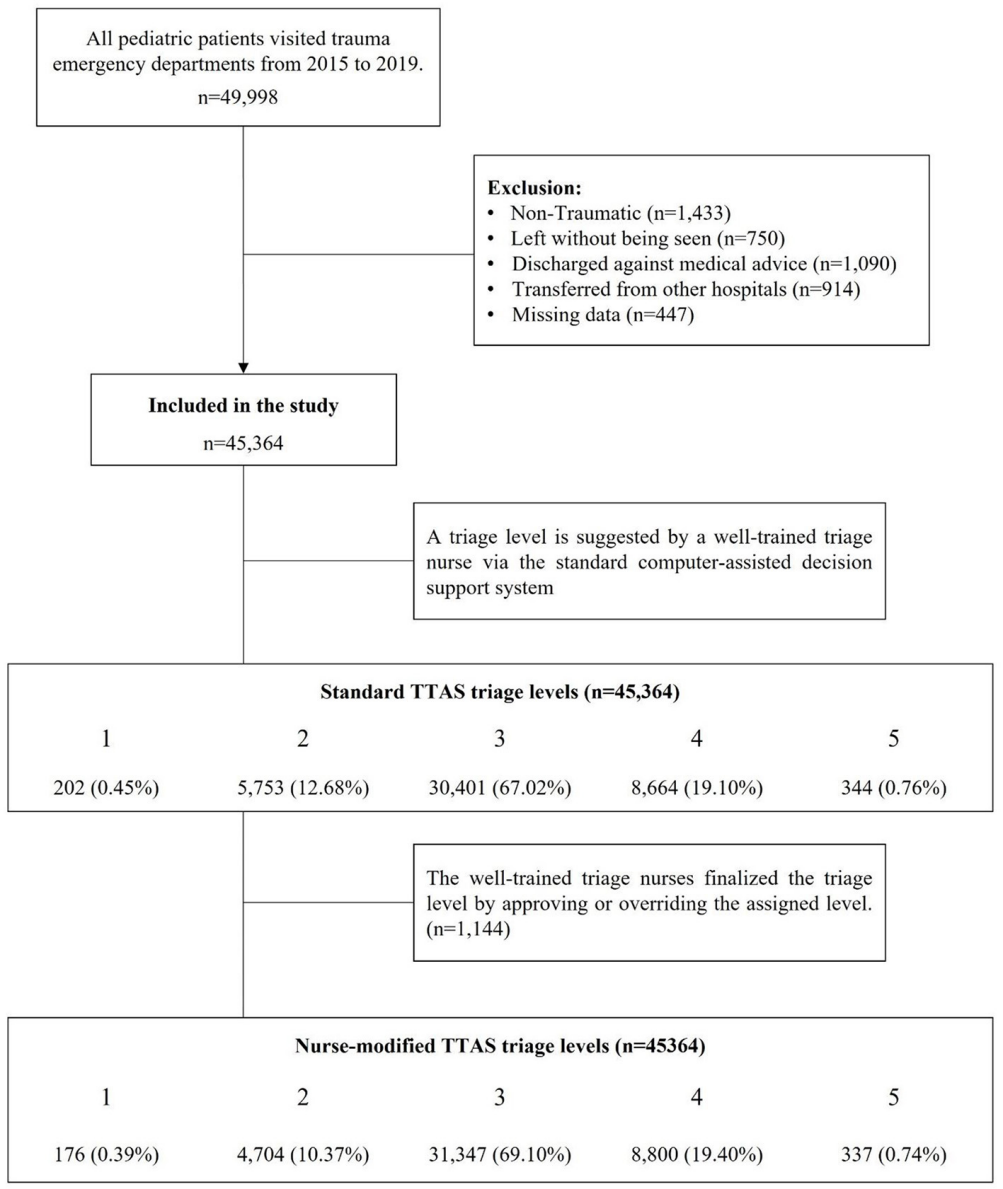
Process for inclusion and exclusion of pediatric traumatic patients visited the EDs, 2015 to 2019. TTAS, Taiwan Triage and Acuity Scale; ED, emergency department.

From January 2015 to December 2019, patients under 18 years of age with traumatic presentations who visited the EDs were included in the analysis. We excluded pediatric patients seeking medical advice due to surgical complications, patients with or without incomplete triage acuity, and those left without being seen or discharged against medical advice. Patients transferred from other hospitals were also excluded because they had already been treated and diagnosed before arrival. The inclusion and exclusion criteria are presented in Figure 1.

### Taiwan triage and acuity scale

The triage nurses performed a short assessment and triaged patients using the TTAS, which retains most features of the Canadian triage and acuity scale (CTAS) but includes some major modifications, such as (1) shorter reassessment time intervals for acuity levels 2 (0, 10, 30, 60, and 120 min, respectively, for levels 1 to 5). (2) Dividing the chief complaint list into a non-trauma category, comparable to the CTAS, and a trauma category, with the chief complaint list organized by anatomical region and environmental injury. The non-trauma system includes 14 categories and 132 chief complaints, while the trauma system has 15 categories and 47 chief complaints. (3) Explicit threshold levels for hemodynamic stability (e.g., tachycardia/bradycardia (140 bpm/50 bpm) with or without symptoms of shock or a blood pressure <90 mmHg). (4) Revised pain severity determination with the deletion of chronic pain to accommodate cultural expectations in Taiwan.

A triage level of the TTAS was assigned for each patient visiting the ED based on the presenting complaint and first- or second-order modifiers *via* a computer-assisted decision support system (Supplementary Figure 1). First-order modifiers consisted of mechanisms of injury and initial vital signs. Children with high-risk traumatic mechanisms (Supplementary Table 1) would be directly assigned a level of two or above, regardless of good or bad vital signs. Furthermore, if a pediatric patient meets the criteria for major or severe trauma (Supplementary Table 2), the trauma team will be activated immediately. Following first-order modifications, the triage nurse could optimize the triage level based on second-order modifiers, including several particular scenarios such as sexual assault. At the end of the triage process, if triage nurses disagree with the assigned TTAS urgency category, the system can be overruled. The reasons for this modification would be recorded.

### Measurements

We collected demographic information (i.e., sex and age), pre-hospital mode of transport (i.e., ambulance, private vehicle, or other), physiological variables (i.e., Glasgow coma scale (GCS), temperature, HR, RR, and mean arterial pressure), clinical characteristics (i.e., high-energy trauma, trauma team activation, type of injury, the primary location of the injury), clinical outcomes (i.e., discharge from ED, general ward admission, intensive care unit admission, mortality in ED, and overall in-hospital mortality), medical resources, ED length of stay (ED-LOS), triage level based on TTAS, and modification approved by triage nurses whose high acuity was defined as level 1 and 2; low acuity was defined as level 3 to 5. High-energy trauma was defined as the presence or absence of high-risk mechanisms of injury in pediatric patients (Supplementary Table 1). The utilization rate of medical resources was evaluated in terms of total medical expenses. Additionally, we used the need for emergency surgical treatment as a metric to assess triage appropriateness in trauma patients, which was defined as ED discharge to the operating room within 90 min (6). The overall hospitalization was defined to include cases admitted to the general ward and the intensive care unit. Pediatric patients who died in the ED were also included in the admission category, reducing the competing risk. Hospital mortality was defined to include patients who died in the ED, general ward, and intensive care unit for reflecting the severity. The ED-LOS variable was calculated from the time interval between registration and leaving the ED. To elucidate age-related differences in the modification of acuity level, pediatric trauma patients were further analyzed in six subgroups according to previous research (<3 months, 3 months−3 years, 3–6 years, 6–9 years, 9–12 years, and >12 years) (16).

### Outcomes

The primary outcome was the performance of the standard computer-assisted TTAS system in pediatric trauma patients by assessing the prediction accuracy of hospitalization, emergency surgery, medical expenses, and ED-LOS for each triage level. The secondary outcomes were to compare the predictive performance of the TTAS before and after modification by nurses and to explore the causes that contributed to triage nursing adjustment of triage acuity level.

### Primary data analysis

Data were summarized using descriptive statistics, in which categorical variables were expressed as frequency and percentage, and continuous variables were expressed as medians and interquartile ranges (IQR). Triage level was regarded as an independent variable. Multivariate regression models adjusted for sex, hospital level, transportation mode, and age group were used for rates of overall hospitalization, emergency surgery, medical expenses, and ED-LOS to analyze the correlations between each acuity level and clinical outcomes. Log transformation was performed on medical costs and ED-LOS before the multivariate linear regression analysis. To estimate the probability that affected the modification of TTAS in pediatric trauma patients based on their characteristics, statistically significant factors in the univariate logistic regression analysis were used to derive a multivariate logistic regression model. In this process, variables with collinearity between factors were excluded. All multivariate models were assessed using the Hosmer–Lemeshow test, C-statistics, and model fit statistics. All analyses were performed using SAS V.9.4 (SAS, Cary, North Carolina, USA). Statistical significance was defined as *p* < 0.05.

## Results

We reviewed the records of 49,998 pediatric trauma patients under 18 years of age who visited EDs between 2015 and 2019. A total of 45,364 pediatric trauma patients met the inclusion criteria of this study. The distribution of the standard TTAS triage levels from one to five was 202 (0.45%), 5,753 (12.68%), 30,401 (67.02%), 8,664 (19.10%), and 344 (0.76%), respectively. Then, the triage nurse would finalize the triage level by approving or overriding the previously assigned level. The distribution of the nurse-modified TTAS triage levels from one to five was 176 (0.39%), 4,704 (10.37%), 31,347 (69.10%), 8,800 (19.40%), and 337 (0.74%) patients, respectively (Figure 1). In total, 1,144 pediatric patients' triage levels were adjusted, including 1,095 (95.72%) down-triages and 49 (4.28%) up-triages.

### Characteristics of study subjects

The baseline characteristics of the study participants are shown in Table 1. In the cohort, 29,687 (65.44%) pediatric patients were male/boy. More than half of the injured children were sent to medical centers. Regarding age distribution, adolescents (age ≥12 years old) were the largest group (16,460 patients, 36.28%), followed by toddlers (3 months to 3 years old, total of 13,433 patients, 29.61%). Most pediatric patients presented with minor trauma. In addition, 98.83% (*n* = 44,834) had full GCS scores. Only 0.47% (*n* = 212) of the patients experienced high-energy trauma. The trauma team was activated 172 times, which accounted for 0.38% of the study cohort. The triage nurses identified 218 child abuse and 40 sexual assault cases during the triage process. Most of the children experienced blunt trauma (60.87%). Among all patients, 31.12% (*n* = 14,118) visited the EDs for skin lacerations or abrasions. Only 0.94% (*n* = 428) of the patients presented with penetrating injuries. Half of the children were mainly injured in the head or neck (50.77%), 37.77% (*n* = 17,136) had extremity injuries, and 6.93% (*n* = 3,145) experienced multiple-body-part injuries regardless of major or minor trauma. Truncal trauma accounted for 4.52% of cases (*n* = 2,052). For resource utilization and disposition, most children were discharged directly from the EDs. A total of 4.31% (*n* = 1,957) of pediatric patients were admitted to an ordinary ward, whereas 1.09% (*n* = 495) were admitted to the intensive care unit. Only 0.76% (*n* = 344) of patients underwent emergency surgery, and 0.17% (*n* = 79) died in the hospital.

**Table 1:** Demographic in pediatric traumatic patients who visited the EDs from 2015 to 2019.

**Characteristics**	**Total *n* = 45,364**		**Modification by triage nurses**			
			**(–)**		**(+)**	
			***n* = 44,220**		***n* = 1,144**	
Male gender, *n* (%)	29,687	(65.44)	29,057	(65.71)	630	(55.07)
Levels of the hospital, *n* (%)						
Academic medical center	25,838	(56.96)	25,452	(57.56)	386	(33.74)
Regional hospital	12,750	(28.11)	12,268	(27.74)	482	(42.13)
District hospital	6,776	(14.94)	6,500	(14.70)	276	(24.13)
Arrival by ambulance, *n* (%)	4,379	(9.65)	4,332	(9.80)	47	(4.11)
Age group, *n* (%)						
Age < 3 MO	363	(0.80)	314	(0.71)	49	(4.28)
3 MO ≤ Age < 3 Y	13,433	(29.61)	12,537	(28.35)	896	(78.32)
3 Y ≤ Age < 6 Y	5,379	(11.86)	5,264	(11.90)	115	(10.05)
6 Y ≤ Age < 9 Y	3,116	(6.87)	3,095	(7.00)	21	(1.84)
9 Y ≤ Age < 12 Y	6,613	(14.58)	6,586	(14.89)	27	(2.36)
12 Y ≤ Age	16,460	(36.28)	16,424	(37.14)	36	(3.15)
Glasgow Coma Scale, *n* (%)						
15	44,834	(98.83)	43,703	(98.83)	1,131	(98.86)
13–14	287	(0.63)	276	(0.62)	11	(0.96)
9–12	163	(0.36)	161	(0.36)	2	(0.17)
3–8	80	(0.18)	80	(0.18)	0	(0)
Vital sign						
BT, median (IQR)	36.30	(0.7)	36.3	(0.7)	36.3	(0.8)
HR, median (IQR)	101.00	(37)	101	(35)	150	(22.5)
RR, median (IQR)	20.00	(4)	20	(4)	22	(5)
MAP, median (IQR)	90.33	(19)	90.33	(19)	93.33	(34)
High-energy trauma, *n* (%)	212	(0.47)	211	(0.48)	1	(0.09)
Trauma team activation, *n* (%)	172	(0.38)	171	(0.39)	1	(0.09)
Child abuse, *n* (%)	218	(0.48)	218	(0.49)	0	(0)
Sexual assault, *n* (%)	40	(0.09)	40	(0.09)	0	(0)
Type of injuries, *n* (%)						
Blunt trauma	27,612	(60.87)	26,951	(60.95)	661	(57.78)
Penetrating trauma	428	(0.94)	423	(0.96)	5	(0.44)
Skin laceration or abrasion	14,118	(31.12)	13,744	(31.08)	374	(32.69)
Burn	1,845	(4.07)	1,769	(4.00)	76	(6.64)
Other	1,361	(3.00)	1,333	(3.01)	28	(2.45)
The primary location of injuries						
Head or neck	23,031	(50.77)	22,317	(50.47)	714	(62.41)
Thorax or abdomen	2,052	(4.52)	2,031	(4.59)	21	(1.84)
Extremities	17,136	(37.77)	16,816	(38.03)	320	(27.97)
Other or multiple-body-part minor injuries	3,145	(6.93)	3,056	(6.91)	89	(7.78)
Resource utilization						
Emergency surgery, *n* (%)	344	(0.76)	342	(0.77)	2	(0.17)
Expenses (NT), median (IQR)	2,236.00	(2,346)	2,250	(2,362)	1,836	(1,811)
LOS (min), median (IQR)	51.00	(69)	51	(69)	40	(52)
Final disposition, *n* (%)						
Discharge from ED	42,882	(94.53)	41,768	(94.45)	1,114	(97.38)
Admitted to ward	1,957	(4.31)	1,937	(4.38)	20	(1.75)
Admitted to ICU	495	(1.09)	486	(1.10)	9	(0.79)
Mortality in ED	30	(0.07)	29	(0.07)	1	(0.09)
Overall in-hospital mortality	79	(0.17)	78	(0.18)	1	(0.09)

ED, emergency department; BT, body temperature; HR, heart rate; ICU, intensive care unit; RR, respiratory rate; MO, months old; MAP, mean arterial pressure; IQR, interquartile range; ED-LOS, emergency department length of stay; TTAS, Taiwan Triage and Acuity Scale; Y, years; NT, New Taiwan Dollars.

### Performance of the TTAS triage system in pediatric trauma patients

Triage nurses agreed with most of the initial TTAS triage levels (97.48%). There were 1,144 (2.52%) children whose initial triage levels were disagreed with by the triage nurses (Table 1). The distributions and odds of hospitalization, emergency surgery, medical expenses, and ED-LOS at each triage level before and after the modifications are shown in Table 2. In the standard TTAS model, all percentages or median values of the four outcomes showed upward trends with rising acuity levels. In addition, all the odds ratios or β values had similar trends, with a significant statistical difference for each triage level compared to level 3 (Table 2).

**Table 2:** Comparison of effectiveness in pediatric trauma patients between the standardized TTAS and nurse-modification TTAS (*n* = 45,364).

**Triage level**	**Overall hospitalization**						**Emergency surgery**					
	**Standard TTAS**			**N-modified TTAS**			**Standard TTAS**			**N-modified TTAS**		
	**%**	**OR**	**95% CI**	**%**	**OR**	**95% CI**	**%**	**OR**	**95% CI**	**%**	**OR**	**95% CI**
1	44.55	11.89	8.66 to 16.32	51.14	13.49	9.69 to 18.78	16.83	21.14	13.59 to 32.87	19.32	21.69	13.92 to 33.79
2	10.71	3.27	2.91 to 3.67	12.50	3.49	3.10 to 3.91	1.69	4.66	3.58 to 6.08	2.02	4.84	3.71 to 6.31
3	5.59	1.00	–	5.50	1.00	–	0.70	1.00	–	0.68	1.00	–
4	0.88	0.18	0.15 to 0.23	0.90	0.19	0.15 to 0.24	0.01	0.02	0.003 to 0.16	0.01	0.02	0.003 to 0.15
5	0.29	0.08	0.01 to 0.55	0.30	0.08	0.01 to 0.55	0.00	–	–	0.00	–	–
Goodness of fit
AIC*	16,807.26			16,765.35			3,307.66			3,301.96		
C statistics	0.75			0.77			0.84			0.85		
**Triage level**	**Expenses (NT Dollars)**						**ED-LOS**					
	**Standard TTAS**			**N-modified TTAS**			**Standard TTAS**			**N-modified TTAS**		
	**Median (IQR)**	β	**95% CI**	**Median (IQR)**	β	**95% CI**	**Media*****n*** **(IQR)**	β	**95% CI**	**Media*****n*** **(IQR)**	β	**95% CI**
1	7,550.5 (119,313)	1.59	1.46 to 1.72	10,039 (155,388)	1.83	1.69 to 1.96	115 (126)	0.41	0.2776 to 0.53	118.5 (119)	0.42	0.29 to 0.56
2	2,399 (3,100)	0.41	0.38 to 0.43	2,552 (4,210)	0.49	0.46 to 0.52	60 (102)	0.20	0.17 to 0.22	66 (115)	0.24	0.21 to 0.27
3	2,365 (2,528)	–	–	2,348 (2,505)	–	–	55 (74)	–	–	55 (73)	–	–
4	1,876.5 (1,433.5)	−0.32	−0.34 to −0.30	1,874 (1,432.5)	−0.32	−0.35 to −0.30	37 (40)	−0.31	−0.33 to −0.29	37 (40)	−0.31	−0.34 to −0.29
5	1,442.5 (1,022)	−0.61	−0.71 to −0.51	1,418 (964)	−0.64	−0.74 to −0.54	25.5 (31)	−0.60	−0.69 to −0.50	25 (30)	−0.61	−0.71 to −0.51
Goodness of fit
AIC*	120,007.71			119,678.11			121,115.32			121,046.10		
BIC*	120,138.29			119,808.69			121,246.16			121,176.94		

Independent variable, Overall hospitalization (Y/N) and emergency surgery (Y/N); In (Expenses and ED-LOS); ^*^performed with multiple regression analysis adjusted by sex, levels of the hospital, mode of transport, and age group.

OR, odds ratio; CI, confidence interval; IQR, interquartile range; AIC, Akaike information criterion; BIC, Bayesian information criterion; TTAS, Taiwan Triage and Acuity Scale; N-modified, nurse-modified, ED-LOS, emergency department length of stay; NT dollars, New Taiwan Dollars.

### Effectiveness of the nurses' modification

In Table 2, we compared the overall hospitalization rate, emergency surgery rate, medical expenses, and ED-LOS at each triage level between the standard TTAS and nurse-modified TTAS. In the nurse-modified model, the distribution and odds of all four outcomes still showed upward trends with rising triage levels. In addition, after nurses' modification, the proportion of hospitalization and emergency surgery increased in the high-acuity group (levels 1 and 2) and decreased in the low-acuity group (levels 3 to 5); the same was true for expenses and ED-LOS, where the medians became higher or longer in the high-acuity group and reduced in the low-acuity group. The regression model also showed similar changing trends in all four outcomes, with higher odds ratios or β values in the high-acuity group and lower in the low-acuity group. Furthermore, we performed a goodness-of-fit test to evaluate the effectiveness of nurses' modifications. Smaller Akaike information criterion (AIC) and Bayesian information criterion (BIC) values, as well as increased C-statistics, were found in the nurse-modified model for all four outcomes. This suggests that better predictive performance was achieved after modification (Table 2).

### Contributory factors affecting modification

**Figure 2:**
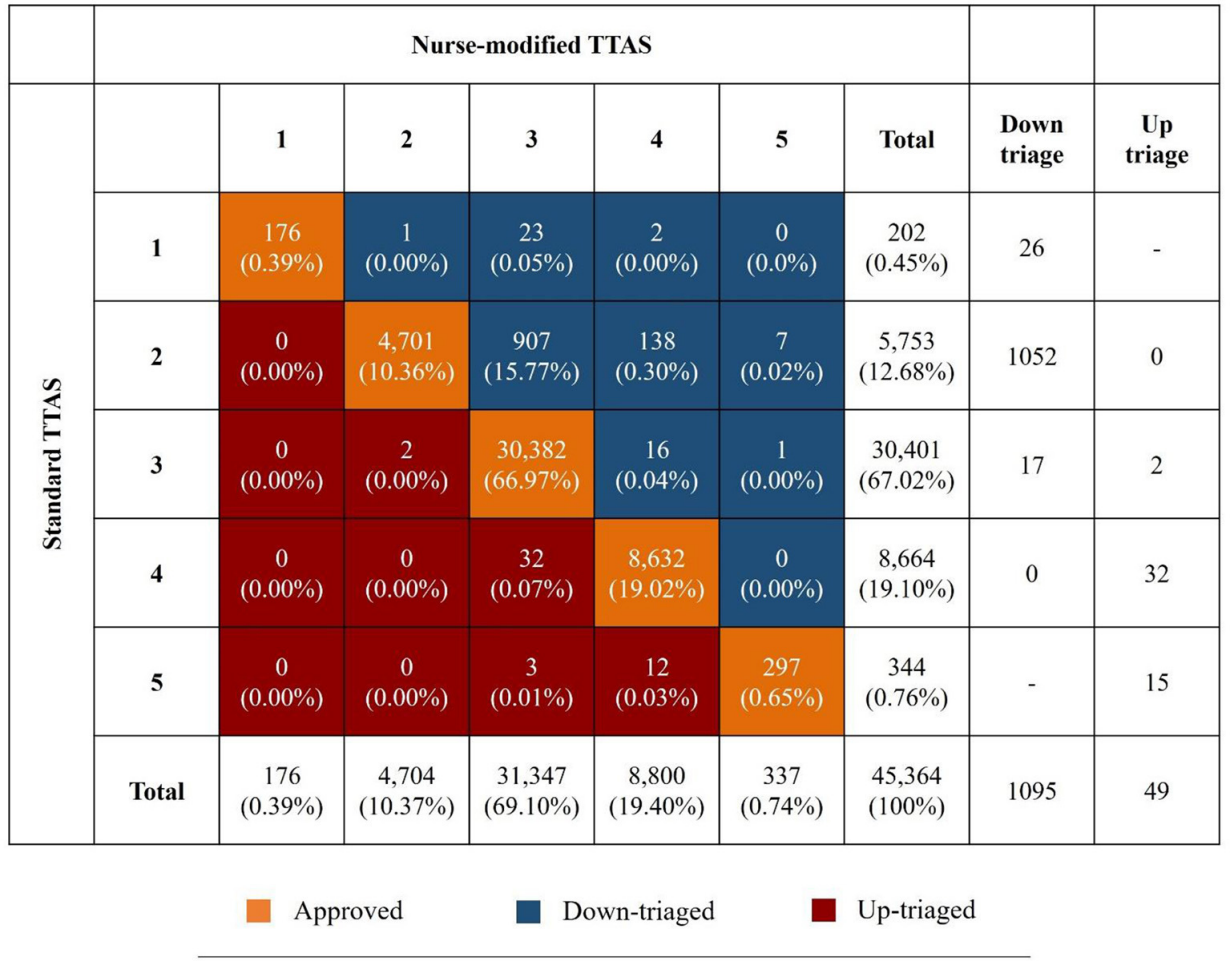
Acuity level distributions of the TTAS before and after triage nurses' modification. TTAS, Taiwan Triage and Acuity Scale.

The acuity level distributions of the TTAS before and after the triage nurses' modifications are shown in Figure 2. Among the children whose triage levels were modified, only 49 cases were uptriaged. A total of 1,095 cases were down-triaged; most were initially assigned to level 2 (91.96%, 1,052 of 1,144). We classified three major subjective reasons for level modifications recorded in the electronic database by the triage nurses, which were (1) the children were assigned a higher triage level for abnormal HR, RR, or saturation, but having a good appearance and minor trauma mechanism (total 1,083 cases, 94.67%). Most of them were considered due to the anxious and irritable emotional responses to the unfamiliar environments (*n* = 447) or caused by severe pain (*n* = 80); (2) the children had normal vital signs and were triaged with lower acuity level but presented with acute ill-looking symptoms (*n* = 34, 2.97%); and (3) the children presented with the proper appearance and vital signs and had no high-risk trauma mechanism, but were not consolable (*n* = 15, 1.31%).

**Figure 3:**
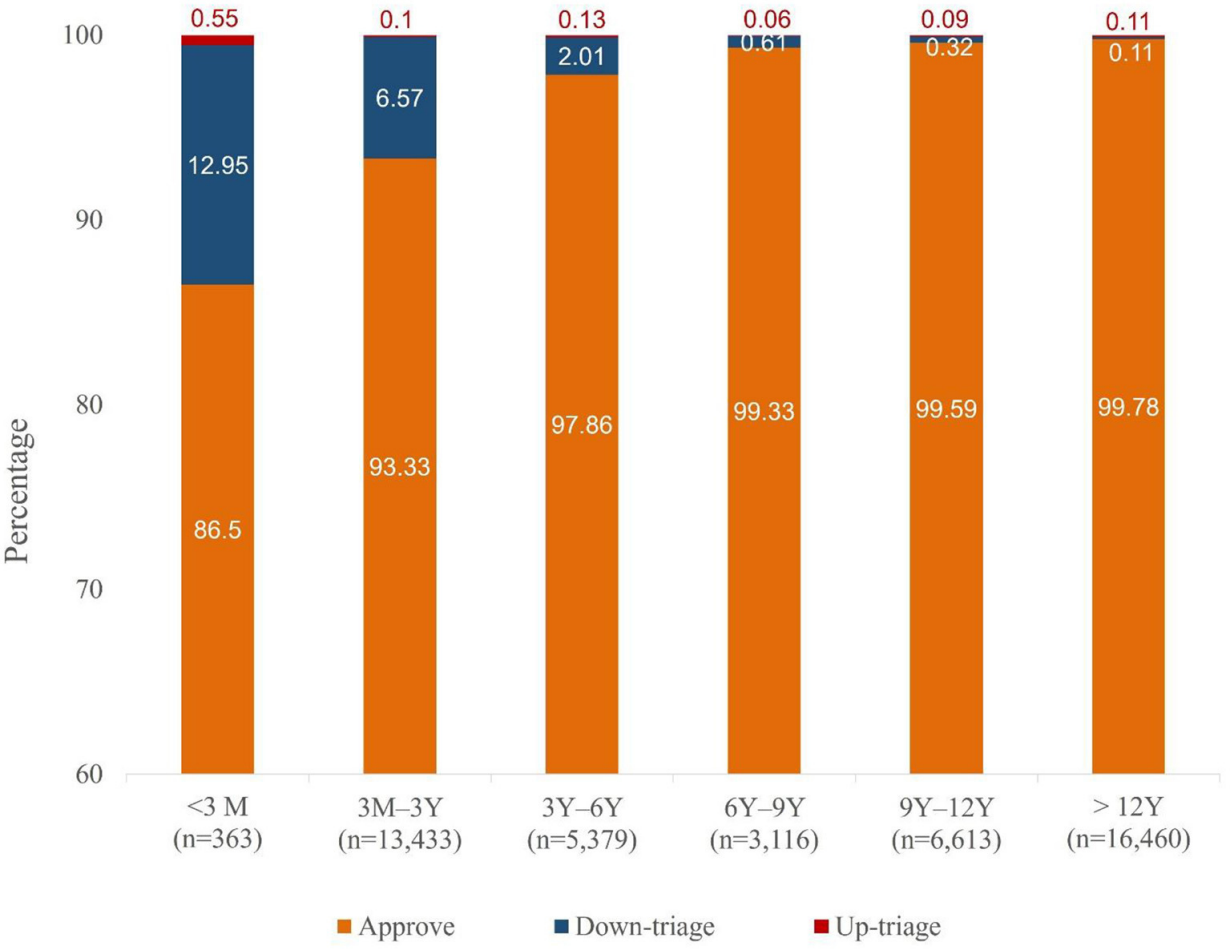
The proportion of nurse-modified group by age. M, months; Y, years.

Regression analysis was used to determine the specific weight of each objective factor that contributed to nurses' modifications. The results of univariate and multivariate logistic analyses are shown in Table 3. In univariate analysis, male sex, hospital level, arrival by ambulance, age group, HR, RR, injury type, and primary location of injuries were significant contributors to modifications. In the multivariate analysis, the odds ratios for nurses' modification tended to become higher as the children's age decreased and particularly showed statistical significance in patients aged <6 years. The odds ratios were 6.71 (*p* < 0.0001) for children aged under 3 months, 4.46 (*p* < 0.0001) for children aged 3 months to 3 years, and 3.17 (*p* < 0.0001) for 3–6 years old. In addition, the level of hospitals, children's HR, RR, and the primary location of injuries were also factors showing a significant correlation with acuity modification. Figure 3 shows the proportion of modification by age. The triage acuity of children under 3 months of age was the most often modified. Most of them (47 of 49 babies) were down-triaged, and only two babies were up-triaged (Supplementary Table 3).

**Table 3:** Univariate and multivariable logistic regression model of associated factors affecting modification (*n* = 45,364).

**Variable**	**Univariate**			**Multivariate**		
	**OR**	**95% CI**	***P-value***	**OR**	**95% CI**	***P-value***
Male gender	0.64	0.57 to 0.72	< 0.0001***	0.90	0.79 to 1.03	0.1377
Levels of hospital						
Academic medical center	Reference			Reference		
Regional hospital	2.59	2.26 to 2.97	< 0.0001***	6.34	5.36 to 7.50	< 0.0001***
District hospital	2.80	2.39 to 3.28	< 0.0001***	8.87	7.31 to 10.76	< 0.0001***
Arrival by ambulance	0.40	0.29 to 0.53	< 0.0001***	0.62	0.44 to 0.86	0.0041*
Age group						
Age < 3 MO	71.18	45.64 to 111.01	< 0.0001***	6.71	4.00 to 11.27	< 0.0001***
3 MO ≤ Age < 3 Y	32.60	23.34 to 45.52	< 0.0001***	4.46	3.01 to 6.49	< 0.0001***
3 Y ≤ Age < 6 Y	9.97	6.85 to 14.51	< 0.0001***	3.17	2.13 to 4.71	< 0.0001***
6 Y ≤ Age < 9 Y	3.10	1.80 to 5.31	< 0.0001***	1.71	0.99 to 2.95	0.0536
9 Y ≤ Age < 12 Y	1.87	1.13 to 3.08	0.0141*	1.32	0.79 to 2.19	0.2846
12 Y ≤ Age	Reference			Reference		
Glasgow Coma Scale						
15	Reference					
13–14	1.54	0.84 to 2.82	0.1622			
9–12	0.48	0.12 to 1.94	0.3027			
3–8	–	–	0.9493			
Vital sign						
BT	1.10	0.99 to 1.22	0.0767			
HR	1.06	1.05 to 1.06	< 0.0001***	1.06	1.05 to 1.06	< 0.0001***
RR	1.12	1.11 to 1.14	< 0.0001***	0.98	0.96 to 0.99	0.0085*
MAP	1.00	0.99 to 1.00	0.1606			
High-energy trauma	–	–	0.9512			
Trauma team activation	–	–	0.9563			
Abuse	0.09	0.01 to 1.42	0.0868			
Rape	0.48	0.03 to 8.03	0.6069			
Type of injuries						
Blunt trauma	Reference			Reference		
Penetrating trauma	0.53	0.23 to 1.23	0.1084	0.90	0.35 to 2.31	0.8287
Skin laceration or abrasion	1.11	0.98 to 1.26	0.1156	1.11	0.96 to 1.29	0.1515
Burn	1.76	1.38 to 2.24	**<**0.0001***	1.00	0.52 to 1.93	0.9969
Other	0.87	0.60 to 1.27	0.4393	0.98	0.62 to 1.53	0.9133
The primary location of injuries						
Head and neck	1.68	1.47 to 1.92	< 0.0001***	1.18	1.01 to 1.38	0.0475*
Thorax or abdomen	0.56	0.36 to 0.86	0.0087*	0.84	0.51 to 1.37	0.4206
Extremities	Reference			Reference		
Other or multiple-body-part minor injuries	1.54	1.21 to 1.95	0.0004**	2.33	1.26 to 4.31	0.0107*

^*^p-value < 0.05; ^**^ *p*-value < 0.001; ^***^ *p*-value < 0.0001; HR, heart rate; RR, respiratory rate; MAP, mean arterial pressure; BT, body temperature; CI, confidence interval; OR, odds ratio; MO, months old; Y, years.

## Discussion

There are many triage tools for acute pediatric trauma in EDs worldwide (7). Some of them have even been used for a long time. To the best of our knowledge, no one has been explicitly validated in pediatric trauma patients. This study validated the TTAS system using four major outcomes: hospitalization, emergency surgery rate, expenses, and ED-LOS. We found this to be an effective triage tool to assist in acuity level determination in acute pediatric trauma patients. Modification of the triage level assigned to pediatric trauma patients based on well-experienced triage nurses' judgment can enhance the predictive performance of the triage system utilized in the quick encounter. According to the overall in-hospital mortality rate, we didn't stratify the mortality rate because most cases suffered from minor injuries and the prediction of the ICU mortality rate mainly relays on other severity scoring systems and the timing of measurement (22, 23). The prediction of the ICU mortality rate is also included in our upcoming research. Besides, we think that ED-LOS can't be the sole parameter used to predict the severity of pediatric trauma patients. For example, a patient may be immediately delivered to the operation room for emergency surgery or suffer from minor injuries and then discharged quickly; in both cases, the ED-LOS was similarly short, but clinical outcomes differed. Therefore, the performance of a triage tool should be validated by utilizing multiple parameters and reliable analyzing methods as we did in this research.

However, 2.52% of the cases were deemed inconsistent with the actual urgency of medical conditions. In this population, we found that 95.72% of cases were over-triaged, which is similar to previous findings in the literature among non-traumatic pediatric patients (10, 16, 24, 25). Over-triaged cases in ED would hinder the fluency of urgent management and compromise the safety of truly critical pediatric trauma patients if the medical resources were occupied (26). Even the over-triaged children were just a small population; the actual number of the children who were delayed being seen might be very large.

Implementing a triage tool to ensure the appropriateness of care for pediatric trauma patients and to remove system barriers is essential for achieving a good quality of care in the emergency workplace (27). We allowed the triage nurses to modify the acuity level at the end of the triage process based on their professional judgment to optimize the triage system. However, few studies have evaluated the effectiveness of triage nurse modifications (14). The CTAS guidelines suggest being aware of overriding the assigned acuity level for patient safety (28). In trauma scenarios, children rarely stay calm at a triage station, and they have just experienced a trauma event, enduring vast physiological and psychological stress. It is difficult for an injured child to cooperate with the triage nurses. Therefore, it is reasonable to allow triage nurses to modify the acuity levels of pediatric trauma patients based on their experience. Takahashi et al. conducted a retrospective analysis of the triage data of 37,961 pediatric patients. They found that level modification based on nurses' subjective experiences could make the triage scale more consistent with hospitalization rates (14). However, it was just a single-center study with only one outcome for assessing its effectiveness, without comprehensive statistical analysis. In our study, we observed that level modification at the end of the triage process could optimize the performance of the triage system by increasing C-statistics and decreasing the values of AIC and BIC, which can aid in predicting not only hospitalization but also emergency surgery, medical expenses, and ED-LOS.

To improve the reliability and validity of the pediatric trauma triage system, it is necessary to determine the factors contributing to inappropriate triage. Prior studies conducted in non-traumatic children have reported that the major reason was the children's emotional reaction to the discomfort or noisy ED environments, which usually cause abnormal vital signs, resulting in over-triage (14–16), especially for children below 6 years of age who are usually difficult to appease and communicate with (14). In our study, we observed similar findings in pediatric patients with trauma. Among the children whose acuity levels were modified, 95.72% were initially over-triaged due to abnormal vitals, and 92.65% were under 6 years. The multivariable regression model also showed that HR, RR, and younger age were the main contributors to nurses' modification. Except for the emotional reaction in young children, another possible explanation might be that all the triage systems were initially designed for adult patients, without specific vital sign criteria for children, and then developed for pediatric triage (29, 30). The anatomy and physiology in older kids are closer to that of adults. In contrast, younger children tend to have higher heart and breathing rates than adults. It is suggested that the vital sign modifiers can be down-weighted in determinations of the acuity levels of younger age groups in pediatric trauma patients. Besides, experienced triage nurses who have been well trained for precise evaluation of the pediatric assessment triangle, namely pediatric general appearance, respiration, and circulation, can assist the effectiveness of the triage tool by appropriately modifying acuity level according to clinical presentations among pediatric trauma patients.

In addition, the way of arrival by ambulance also showed a significant correlation with triage modification. Children sent to EDs by ambulance were less likely to be re-assigned to a triage level. This may be because these children had a higher chance of having severe injuries before being approached; therefore, they had a lower possibility of over-triage. In addition, there was a significant correlation between hospital level and triage modification in our study. The odds ratio of triage modification was highest in district hospitals, followed by regional hospitals and academic medical centers. This might be related to a different experience of the disease and acuity patterns of pediatric ED visits corresponding to the role of varying levels of the hospital in the healthcare system. For example, triage nurses who worked in medical centers had more experience and encounter with critically injured children; therefore, they had fewer pediatric trauma patients with over-triage and a lower odds ratio for triage modification. The GCS score has been revealed in many studies as a significant predictor of outcomes in pediatric trauma (31, 32). Therefore, in contrast to tachycardia or other vital signs, the GCS score showed no association with the nurses' revision of the triage level. A lower GCS score can reliably reflect the patients' critical conditions. In other words, the more serious the injury, the lower the possibility of triage-level modification.

In our study, regression analysis revealed that the injury sites had a significant association with triage modifications. Over 60% of the children whose triage level was modified presented to the EDs with head or neck injuries in our cohort. Most were down-triaged by triage nurses. Severe head or neck injuries always cause severe disability; however, over 90% of head injuries in children experience minor trauma and do not require specific treatment (33, 34). This might suggest that the TTAS triage rule for head or neck injuries should be revised to fit the actual severity and can be assisted by experienced triage nurses' modification if the general appearance evaluation doesn't match the acuity assigned by the triage system. In contrast, thoracic and abdominal injuries showed no statistical significance for acuity modifications in regression analysis. This suggests that TTAS is sufficient to predict the outcomes of children with torso trauma. Unlike head injury, torso trauma is not a common complaint among children; however, it can easily result in severe damage. Children usually lack complete ossification of the ribs and sternum and have less fat or soft tissue to protect their internal organs. The diaphragm is also more horizontal than in adults, which pushes the liver, spleen, and kidneys below the rib edge (35). Therefore, children with thoracic or abdominal trauma would have higher risks of severe injuries, tending to have higher acuity levels and fewer possibilities to be down-triaged. Thus, children with truncal trauma are less likely to undergo triage modifications. Further studies are needed to determine the role of injury sites in triage rules.

### Strength and limitation

Our results provide the first national evidence to validate a triage system in pediatric trauma using a multi-center database with highly complete and colossal sample size. Besides, we utilized AIC, BIC, and C statistics to address different aspects of model fit and measure the prediction performance of TTAS. The study can also add more understanding about how experienced triage nurse modification can assist the effectiveness of current triage tools on pediatric trauma patients. Despite our significant findings, this retrospective cohort study is not without limitations. First, we had incomplete or missing data; however, the percentage was 0.89% for the variables included in the analysis. Second, we could not obtain the detailed mechanism of injuries from the database because they were recorded as nonstructural descriptions. By analyzing the detailed mechanism of injuries, we can determine how those severe damages have occurred, improving the identification accuracy of triage nurses' risk cognition, and raising parents' awareness of the importance of injury prevention. Third, this study was conducted in Taiwan's medical environment, where national health insurance policies promised good accessibility of medical care and relatively low prices, leading to more non-urgent ED visits. Therefore, the results may vary depending on the medical environments and situations of other countries.

## Conclusion

The TTAS is a reliable triage tool with high consistency in hospitalization, emergency surgery, medical expenses, and ED-LOS according to the acuity level assigned to pediatric trauma patients. Level modification at the end of the triage process by well-trained triage nurses can potentially strengthen the predictive performance of the pediatric trauma triage system by adjusting the triage level of pediatric patients with an emotional reaction to pain or stressful and chaotic ED environments, causing abnormal vital signs. The primary objective contributors to acuity modification were children's HR, RR, age under six, and the primary location of injuries. Further external validation is required to determine its role in pediatric trauma worldwide. Besides, we need to construct a data-driven revision cycle and design training curriculums and handouts for triage stakeholders to develop a sustainable and valid five-level triage system in pediatric trauma.

## Data availability statement

The original contributions presented in the study are included in the article/Supplementary material, further inquiries can be directed to the corresponding authors.

## Ethics statement

The studies involving human participants were reviewed and approved by Chang Gung Medical Foundation Institutional Review Board (IRB number 2004110008). Written informed consent from the participants' legal guardian/next of kin was not required to participate in this study in accordance with the National Legislation and the Institutional requirements.

## Author contributions

Conceptualization and investigation: T-TL and Y-CC. Data curation and writing-original draft: T-TL. Formal analysis: T-TL, C-TC, and C-HC. Methodology: T-TL, M-JJ, and Y-CC. Resources and supervision: M-JJ and Y-CC. Validation and writing-review and editing: C-TC, C-PH, C-HC, and C-JN. Visualization: T-TL, C-TC, and Y-CC. All authors contributed to the article and approved the submitted version.

## Conflict of interest

The authors declare that the research was conducted in the absence of any commercial or financial relationships that could be construed as a potential conflict of interest.

## Publisher's note

All claims expressed in this article are solely those of the authors and do not necessarily represent those of their affiliated organizations, or those of the publisher, the editors and the reviewers. Any product that may be evaluated in this article, or claim that may be made by its manufacturer, is not guaranteed or endorsed by the publisher.

## References

[1] Roth GA Abate D Abate KH Abay SM Abbafati C Abbasi N. Global, regional, and national age-sex-specific mortality for 282 causes of death in 195 countries and territories, 1980–2017: a systematic analysis for the Global Burden of Disease Study 2017. Lancet. (2018) 392:1736–88. 10.1016/S0140-6736(18)32203-7 30496103 PMC6227606

[2] World Health Organization. WHO Guidelines Approved by the Guidelines Review Committee. In: Peden M Oyegbite K Ozanne-Smith J Hyder AA Branche C Rahman A et al., editors. World Report on Child Injury Prevention. Geneva: World Health Organization (2008). 26269872

[3] Fitzgerald M Cameron P Mackenzie C Farrow N Scicluna P Gocentas R. Trauma resuscitation errors and computer-assisted decision support. Arch Surgery. (2011) 146:218-25. 10.1001/archsurg.2010.333 21339436

[4] Kondo Y Abe T Kohshi K Tokuda Y Cook EF Kukita I. Revised trauma scoring system to predict in-hospital mortality in the emergency department: Glasgow Coma Scale, age, and systolic blood pressure score. Crit Care. (2011) 15:R191. 10.1186/cc10348 21831280 PMC3387633

[5] Wärnberg Gerdin L Khajanchi M Kumar V Roy N Saha ML Soni KD. Comparison of emergency department trauma triage performance of clinicians and clinical prediction models: a cohort study in India. BMJ Open. (2020) 10:e032900. 10.1136/bmjopen-2019-032900 32075827 PMC7044989

[6] McGaha P. 2nd Garwe T Johnson J Stewart K Sarwar Z Letton RW. So you need a surgeon? Need for surgeon presence as an alternative metric to predict outcomes and assess triage in the pediatric trauma population. J Pediatr Surg. (2020) 55:2124–7. 10.1016/j.jpedsurg.2019.10.055 31761456 PMC9587694

[7] Magalhaes-Barbosa MC Robaina JR Prata-Barbosa A Lopes CS. Reliability of triage systems for pediatric emergency care: a systematic review. Emerg Med J. (2019) 36:231–8. 10.1136/emermed-2018-207781 30630838

[8] de Magalhaes-Barbosa MC Robaina JR Prata-Barbosa A Lopes CS. Validity of triage systems for pediatric emergency care: a systematic review. Emerg Med J. (2017) 34:711–9. 10.1136/emermed-2016-206058 28978650

[9] van der Sluijs R van Rein EAJ Wijnand JGJ Leenen LPH van Heijl M. Accuracy of pediatric trauma field triage: a systematic review. JAMA Surg. (2018) 153:671–6. 10.1001/jamasurg.2018.1050 29799916

[10] Cooper RJ Schriger DL Flaherty HL Lin EJ Hubbell KA. Effect of vital signs on triage decisions. Ann Emerg Med. (2002) 39:223–32. 10.1067/mem.2002.121524 11867973

[11] Van den Bruel A Haj-Hassan T Thompson M Buntinx F Mant D. European research network on recognising serious infection I. Diagnostic value of clinical features at presentation to identify serious infection in children in developed countries: a systematic review. Lancet. (2010) 375:834–45. 10.1016/S0140-6736(09)62000-6 20132979

[12] Thomas DO. Special considerations for pediatric triage in the emergency department. Nurs Clin North Am. (2002) 37:145–59. 10.1016/S0029-6465(03)00090-2 11818269

[13] Christ M Grossmann F Winter D Bingisser R Platz E. Modern triage in the emergency department. Dtsch Arztebl Int. (2010) 107:892–8. 10.3238/arztebl.2010.0892 21246025 PMC3021905

[14] Takahashi T Inoue N Shimizu N Terakawa T Goldman RD. 'Down-triage' for children with abnormal vital signs: evaluation of a new triage practice at a pediatric emergency department in Japan. Emerg Med J. (2016) 33:533–7. 10.1136/emermed-2015-204968 27044947

[15] Chang YC Ng CJ Wu CT Chen LC Chen JC Hsu KH. Effectiveness of a five-level pediatric triage system: an analysis of resource utilization in the emergency department in Taiwan. Emerg Med J. (2013) 30:735–9. 10.1136/emermed-2012-201362 22983978 PMC3756519

[16] Chang YC Ng CJ Wu CT Chen LC Chen JC Hsu KH. Pediatric overtriage as a consequence of the tachycardia responses of children upon ED admission. Am J Emerg Med. (2015) 33:1–6. 10.1016/j.ajem.2014.09.037 25445860

[17] Burd RS Jang TS Nair SS. Evaluation of the relationship between mechanism of injury and outcome in pediatric trauma. J Trauma. (2007) 62:1004–14. 10.1097/01.ta.0000221555.01704.c9 17426560 PMC4969570

[18] Stafford PW Blinman TA Nance ML. Practical points in evaluation and resuscitation of the injured child. Surg Clin North Am. (2002) 82:273–301. 10.1016/S0039-6109(02)00006-3 12113366

[19] Hohenhaus SM Travers D Mecham N. Pediatric triage: a review of emergency education literature. J Emerg Nurs. (2008) 34:308–13. 10.1016/j.jen.2007.06.022 18640410

[20] van Veen M Steyerberg EW Ruige M van Meurs AH Roukema J van der Lei J. Manchester triage system in pediatric emergency care: prospective observational study. BMJ. (2008) 337:a1501. 10.1136/bmj.a1501 18809587 PMC2548283

[21] Shao SC Chan YY Kao Yang YH Lin SJ Hung MJ Chien RN. The Chang Gung Research Database-A multi-institutional electronic medical records database for real-world epidemiological studies in Taiwan. Pharmacoepidemiol Drug Saf. (2019) 28:593–600. 10.1002/pds.4713 30648314

[22] Awad A Bader-El-Den M McNicholas J Briggs J El-Sonbaty Y. Predicting hospital mortality for intensive care unit patients: time-series analysis. Health Informatics J. (2020) 26:1043–59. 10.1177/1460458219850323 31347428

[23] Sprung CL Geber D Eidelman LA Baras M Pizov R Nimrod A. Evaluation of triage decisions for intensive care admission. Crit Care Med. (1999) 27:1073-9. 10.1097/00003246-199906000-00021 10397207

[24] Wier LM Yu H Owens PL Washington R. Overview of children in the emergency department, 2010: statistical brief #157. Healthcare Cost and Utilization Project (HCUP). Stat Brief. (2006). 24006551

[25] Eitel DR Travers DA Rosenau AM Gilboy N Wuerz RC. The emergency severity index triage algorithm version 2 is reliable and valid. Acad Emerg Med. (2003) 10:1070–80. 10.1197/S1069-6563(03)00350-6 14525740

[26] Considine J Ung L Thomas S. Triage nurses' decisions using the National Triage Scale for Australian emergency departments. Accid Emerg Nurs. (2000) 8:201–9. 10.1054/aaen.2000.0166 11760322

[27] Ageron FX Porteaud J Evain JN Millet A Greze J Vallot C. Effect of under triage on early mortality after major pediatric trauma: a registry-based propensity score matching analysis. World J Emerg Surg. (2021) 16:1. 10.1186/s13017-020-00345-w 33413465 PMC7791780

[28] Bullard MJ Musgrave E Warren D Unger B Skeldon T Grierson R. Revisions to the Canadian Emergency Department Triage and Acuity Scale (CTAS) guidelines 2016. CJEM. (2017) 19:S18–27. 10.1017/cem.2017.365 28756800

[29] Tang N Stein J Hsia RY Maselli JH Gonzales R. Trends and characteristics of US emergency department visits, 1997-2007. JAMA. (2010) 304:664–70. 10.1001/jama.2010.1112 20699458 PMC3123697

[30] Mora MC Veras L Burke RV Cassidy LD Christopherson N Cunningham A. Pediatric trauma triage: a pediatric trauma society research committee systematic review. J Trauma Acute Care Surg. (2020) 89:623–30. 10.1097/TA.0000000000002713 32301877

[31] Huang YT Huang YH Hsieh CH Li CJ Chiu IM. Comparison of injury severity score, glasgow coma scale, and revised trauma score in predicting the mortality and prolonged ICU stay of traumatic young children: a cross-sectional retrospective study. Emerg Med Int. (2019) 2019:5453624. 10.1155/2019/5453624 31885926 PMC6914995

[32] Hannan EL Farrell LS Meaker PS Cooper A. Predicting inpatient mortality for pediatric trauma patients with blunt injuries: a better alternative. J Pediatr Surg. (2000) 35:155–9. 10.1016/S0022-3468(00)90001-0 10693657

[33] Finch CF Clapperton AJ McCrory P. Increasing incidence of hospitalization for sport-related concussion in Victoria, Australia. Med J Aust. (2013) 198:427–30. 10.5694/mja12.11217 23641993

[34] Kim HB Kim DK Kwak YH Shin SD Song KJ Lee SC. Epidemiology of traumatic head injury in Korean children. J Korean Med Sci. (2012) 27:437–42. 10.3346/jkms.2012.27.4.437 22468109 PMC3314858

[35] Galvagno SM Jr. Nahmias JT Young DA. Advanced Trauma Life Support(^®^) update 2019: management and applications for adults and special populations. Anesthesiol Clin. (2019) 37:13-32. 10.1016/j.anclin.2018.09.009 30711226

