# Supplementary material for: Validation of a five-level triage system in pediatric trauma and the effectiveness of triage nurse modification: A multi-center cohort analysis
Source: Front Med (Lausanne). 2022 Nov 1;9:947501. doi: 10.3389/fmed.2022.947501 (PMC9664936; doi:10.3389/fmed.2022.947501)
Supplement: Supplementary file 1 [file Data_Sheet_1.pdf]

Supplementary Material

1 Supplementary Material: Figures

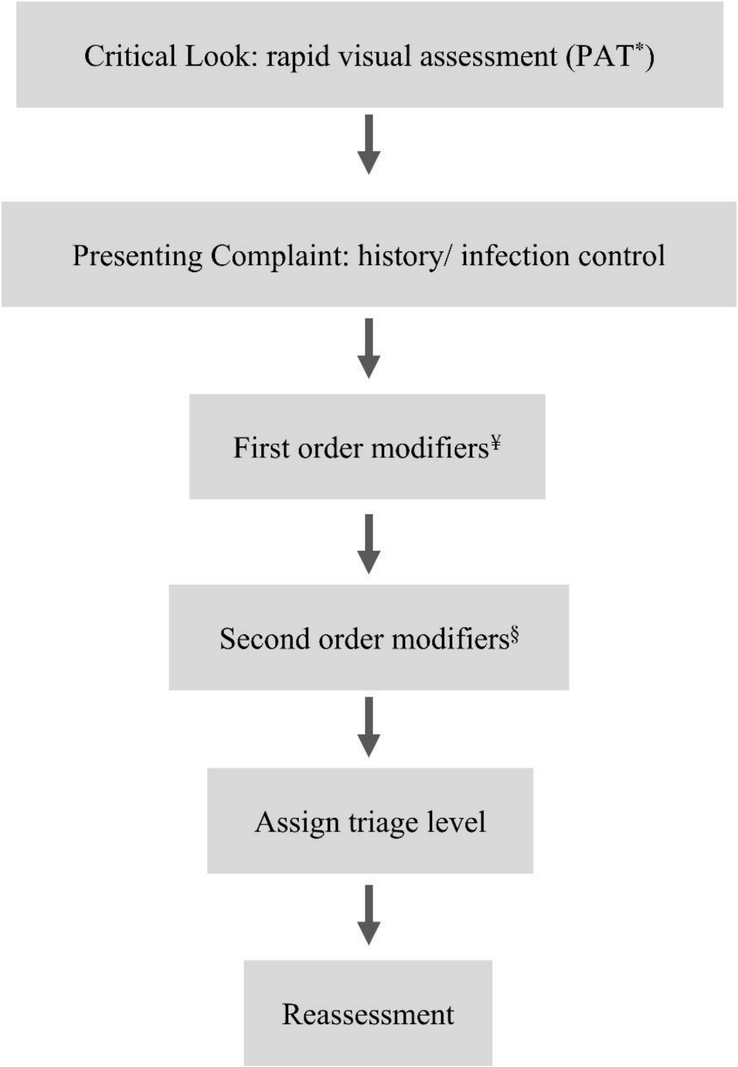

Supplementary Figure 1. The Triaging Process of TTAS

[*]PAT, Pediatric assessment triangle; TTAS, Taiwan Triage and Acuity Scale.

[¥]First-order modifiers: first-step physiological assessments (vital signs), second-step pain severity, mechanism of injury.

[§]Second-order modifiers: Specific to a limited number of complaints, may be required to supplement first-order modifiers to ensure that the patient is assigned an appropriate acuity level.

2 Supplementary Materials: Tables

Supplementary Table 1. The Definition of the high-risk mechanism for injury in TTAS

**Mechanism of injury**	**TTAS Level 2**
General Trauma	1. **MVC**: ejection from vehicle, rollover, extrication time > 20 minutes, significant intrusion into passenger's space, death in the same passenger compartment, impact > 40 km/h (unrestrained) or impact > 60 km/h(restrained).<br>2. **MCC**: where impact with a car > 30 km/h, especially if the rider is separated from the motorcycle.<br>3. **Pedestrian or bicyclist**: run over or struck by a vehicle at > 10 km/h.<br>4. **Fall**: from two times the height of the patient.<br>5. **Penetrating injury**: head, neck, torso, or extremities proximal to elbow and knee.<br>6. **Gunshot**
Head Trauma	1. **MVC**: ejection from the vehicle, unrestrained passenger striking head on the windshield.<br>2. **Pedestrian**: struck by a vehicle.<br>3. **Fall**: from two times the height of the patient.<br>4. **Assault**: with a blunt object other than fist or feet.
Neck Trauma	1. **MVC**: ejection from the vehicle, rollover, high speed (esp. if the driver is unrestrained).<br>2. **MCC**: where impact with a car > 30 km/h, especially if the rider is separated from the motorcycle.<br>3. **Fall**: from two times the height of the patient.<br>4. **Axial load to the head**

MVC, motor vehicle collision; MCC, motorcycle crashes; TTAS, Taiwan Triage and Acuity Scale.

Supplementary Table 2. The Criteria of Trauma Team Activation

Unstable vital signs	1. Unconscious: GCS ≤ 12<br>2. Unstable hemodynamic: SBP < 90 mmHg<br>3. Respiratory distress: RR ≧ 30/min or < 10 min or pulse oximeter < 90%
Major trauma to the mechanism	1. Fall: > 6 meter or > 2 floors<br>2. Run over by vehicles or heavy objects<br>3. Ejection from vehicle<br>4. Death in the same passenger compartment
High-risk site of injury	1. Penetrating trauma of head, neck, or trunk<br>2. Burn injury on the face, second-degree and above

GCS: Glasgow coma scale; SBP: Systolic blood pressure; RR: Respiratory rate

Supplementary Table 3. Demographic in pediatric traumatic patients who had the modification of triage acuity (n=1,144)

**Characteristics**	**Up-triage (n=49)**		**Down-triage (n=1095)**	
Male gender, n (%)	36	(73.47)	594	(54.25)
Levels of the hospital, n (%)				
Academic medical center	14	(28.57)	372	(33.97)
Regional hospital	9	(18.37)	473	(43.20)
District hospital	26	(53.06)	250	(22.83)
Arrival by ambulance, n (%)	2	(4.08)	45	(4.11)
Age group, n (%)				
Age < 3 MO	2	(4.08)	47	(4.29)
3MO ≤ Age < 3Y	14	(28.57)	882	(80.55)
3Y ≤ Age < 6Y	7	(14.29)	108	(9.86)
6Y ≤ Age < 9Y	2	(4.08)	19	(1.74)
9Y ≤ Age < 12Y	6	(12.24)	21	(1.92)
12Y ≤ Age	18	(36.73)	18	(1.64)
Glasgow Coma Scale, n (%)				
15	48	(97.96)	1083	(98.90)
13–14	1	(2.04)	10	(0.91)
9–12	0	(0)	2	(0.18)
3–8	0	(0)	0	(0)
Vital sign				
BT, median (IQR)	36.30	(0.70)	36.30	(0.80)

HR, median (IQR)	103.50	(32.00)	151.00	(22.00)
RR, median (IQR)	18.00	(2.50)	22.00	(6.00)
MAP, median (IQR)*	89.33	(28.00)	93.83	(35.33)
High-energy trauma, n (%)	1	(2.04)	0	(0)
Trauma team activation, n (%)	1	(2.04)	0	(0)
Abuse, n (%)	0	(0)	0	(0)
Rape, n (%)	0	(0)	0	(0)
Type of injuries, n (%)				
Blunt trauma	32	(65.31)	629	(57.44)
Penetrating trauma	1	(2.04)	4	(0.37)
Laceration and Abrasion injury	12	(24.49)	362	(33.06)
Burn	2	(4.08)	74	(6.76)
Other	2	(4.08)	26	(2.37)
The primary location of injuries				
Head or neck	20	(40.82)	694	(63.38)
Thorax or abdomen	1	(2.04)	20	(1.83)
Extremities	26	(53.06)	294	(26.85)
Other or multiple injuries	2	(4.08)	87	(7.95)
Resource utilization				
Emergency surgery, n (%)	0		2	(0.18)
Expenses (NT), median (IQR)	2293.00	(2390.00)	1812.00	(1802.00)
ED-LOS (min), median (IQR)	37.00	(38.00)	40.00	(53.00)
Final disposition, n (%)				
Discharge from ED	48	(97.96)	1066	(97.35)
Admitted to ward	1	(2.04)	19	(1.74)

Admitted to ICU	0	(0)	9	(0.82)
Mortality in ED	0	(0)	1	(0.09)
Mortality in hospital	0	(0)	1	(0.09)

BT, body temperature; HR, heart rate; RR, respiratory rate; MAP, mean arterial pressure; IQR, interquartile range; ED, emergency department; ED-LOS, emergency department-length of stay; MO, months old; Y, year; ICU, intensive care unit; NT, New Taiwan Dollars.

Supplementary Table 4. Univariate and multivariable logistic regression model of associated factors affecting up-triage (n=1,144)

**Variable**	**Univariate**			**Multivariate**		
	**OR**	**95% CI**	***P-value***	**OR**	**95% CI**	***P-value***
Male sex	2.34	1.23 to 4.45	0.0100*	1.826	0.86 to 3.87	0.1160
Levels of hospital						
Academic medical center	Reference			Reference		
Regional hospital	0.51	0.22 to 1.18	0.1151	0.57	0.22 to 1.43	0.2302
District hospital	2.76	1.42 to 5.40	0.0029*	1.46	0.64 to 3.29	0.3675
Arrival by ambulance	0.99	0.23 to 4.22	0.9923			
Age group						
Age < 3 MO	0.04	0.01 to 0.20	<.0001***	0.88	0.15 to 5.22	0.8891
3MO ≤ Age < 3Y	0.02	0.01 to 0.04	<.0001***	0.14	0.05 to 0.45	0.0009**
3Y ≤ Age < 6Y	0.07	0.02 to 0.18	<.0001***	0.22	0.07 to 0.69	0.0098*
6Y ≤ Age < 9Y	0.11	0.02 to 0.18	0.0057*	0.27	0.06 to 1.33	0.1080
9Y ≤ Age < 12Y	0.29	0.09 to 0.87	0.0281*	0.45	0.13 to 1.62	0.2223
12Y ≤ Age	Reference			Reference		
Glasgow Coma Scale						
15	Reference					
13–14	2.26	0.28 to 17.99	0.4423			
9–12	-	-	0.9896			
3–8	-	-	-			
Vital sign						
BT	0.83	0.52 to 1.35	0.4611			
HR	0.95	0.94 to 0.96	<.0001***	0.98	0.96 to 0.99	0.0008**

RR	0.71	0.64 0.80	<.0001***	0.82	0.72 to 0.93	0.0021*
MAP	0.99	0.98 1.01	0.4392			
High-energy trauma	-	-	-			
Trauma team activation	-	-	-			
Abuse	-	-	-			
Rape	-	-	-			
Type of injuries						
Blunt trauma	1.54	0.41 to 5.74	0.5213			
Penetrating trauma	9.94	0.91 to 108.31	0.0595			
Laceration and Abrasion injury	1.03	0.26 to 4.12	0.9694			
Burn	Reference			Reference		
Other	2.81	0.45 to 17.53	0.2684			
The primary location of injuries						
Head and neck	0.33	0.18 to 0.59	0.0002**			
Thorax or abdomen	0.57	0.07 to 4.38	0.5851			
Extremities	Reference			Reference		
Other or multiple injuries	0.26	0.06 to 1.12	0.0701			

*p value<.05; **p value<.001; ***p value<.0001.

HR: heart rate; RR: respiratory rate; MAP: mean arterial pressure; Y, year; MO, months old; BT, body temperature; CI, confidence interval; OR, odds ratio.
